# Comparative analysis of volatiles difference of Yunnan sun-dried Pu-erh green tea from different tea mountains: Jingmai and Wuliang mountain by chemical fingerprint similarity combined with principal component analysis and cluster analysis

**DOI:** 10.1186/s13065-016-0159-y

**Published:** 2016-03-10

**Authors:** Yuanshuang Wu, Shidong Lv, Chen Wang, Xuemei Gao, Jiangbing Li, Qingxiong Meng

**Affiliations:** Faculty of Life Science and Technology, Kunming University of Science and Technology, Kunming, 650500 Yunnan People’s Republic of China; Kunming Grain and Oil and Feed Product Quality Inspection Center, Kunming, 650118 Yunnan People’s Republic of China

**Keywords:** Pu-erh green tea, Gas chromatography-mass spectrometry, Chemical fingerprint similarity, Principal component analysis, Cluster analysis

## Abstract

**Background:**

Modern instrumental analysis technology can provide various chemical data and information on tea samples. Unfortunately, it remains difficult to extract the useful information. We describe the use of chemical fingerprint similarities, combined with principal component and cluster analyses, to distinguish and recognize Pu-erh green teas, which from two tea mountains, Wuliang and Jingmai, in the Pu-erh district of Yunnan province. The volatile components of all 20 Pu-erh green teas (10 Wuliang and 10 Jingmai teas) were extracted and identified by headspace solid-phase micro extraction (HS-SPME) combined with gas chromatography-mass spectrometry (GC-MS).

**Results:**

Sixty-three volatiles (including alcohols, hydrocarbons, ketones, and aldehydes) were identified in the 20 Pu-erh green teas, and differences in compound compositions between them were also observed. Through fingerprint similarity, combined with principal component and cluster analyses, the 20 Pu-erh green teas were differentiated successfully based on their volatile characteristics.

**Conclusions:**

This study demonstrates that the GC-MS combined with chemical fingerprint and unsupervised pattern recognition method is suitable for the investigation of the volatile profiling and evaluating the quality and authenticity of teas related to the different origins.

**Graphical abstract:**
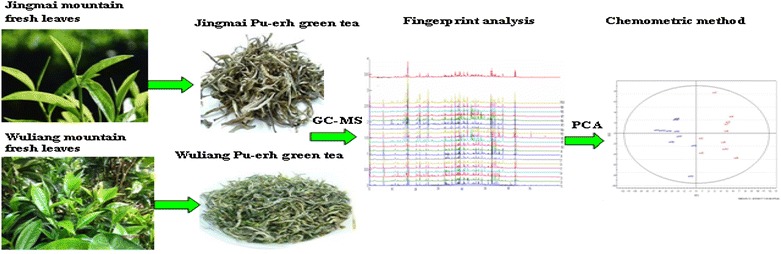
Differentiate Pu-erh green teas from different tea mountains by using chemical fingerprint similarity and multivariate statistical methods

**Electronic supplementary material:**

The online version of this article (doi:10.1186/s13065-016-0159-y) contains supplementary material, which is available to authorized users.

## Background

Pu-erh tea has been produced from the big-leaf species of tea trees in China’s Yunnan province for 1700 years [1]. Pu-erh tea can be further divided into green tea and ripe tea based on different processing techniques. Pu-erh green tea retains the original color of the tea leaves and is not fermented during processing, whereas ripe tea is fermented before drying [2]. Pu-erh green tea is very popular and attracts numerous domestic and foreign consumers owing to its distinct flavor and potential biological and pharmaceutical properties, such as anticancer, hypolipidemic, antioxidant, and oxygen free radical elimination [3].

Yunnan’s forests and smallholder agro-ecosystems result in a diversity of teas, including dozens of wild relatives and hundreds of cultivars and local varieties [4]. Tea in Yunnan grows in forests (mountain), agro-forests, mixed crop fields, and terrace plantations. Forest (mountain) tea generally includes tea trees that are wild, were sparsely planted in forests, or were cultivated and have become feral. Because of the absence of artificial watering, spraying pesticides and fertilizing, forest (mountain) tea usually is better adapted ecologically and environmentally and is of better quality, has more abundant minerals, and a stronger fragrance and taste, compared with tableland teas. However, the forest tea is relatively more expensive than tableland tea owing to its low yield and difficulty in harvesting.

Jingmai and Wuliang mountains are two representative tea mountains in Yunnan province. Jingmai mountain is in the Lancang county of the Pu-erh district. Jingmai ancient tea gardens are regarded as the provenance of the well-known Pu-erh tea. Jingmai tea has an intense and persistent fragrance, and its tea infusion has a relatively weak bitter and strong astringent taste. Wuliang mountain, which is in the Jingdong county of Pu-erh district, has rich resources of aged tea trees, and is one of the largest Pu-erh tea producing mountains. The Wuliang tea infusion has a relatively strong bitter and weak astringent taste, and its fragrance is rich and lasting. Benefiting from different geographical locations and natural resources, the Pu-erh teas produced from these two tea mountains exhibit different quality characteristics. The differences in altitudes, climates, rainfalls, and soil mineral structures between the mountains are potential factors in influencing tea quality, and even results in notable fragrance differences that form special “mountain incense”. Artificial cultivation of tea trees leads to the absorption of more superficial soil nutrients, and the application of unified fertilizing guidelines and chemical fertilizers can result in the origin-related special fragrance gradually disappearing with storage time [5, 6].

Because Pu-erh green tea from different tea mountains has different quality characteristics, their prices can vary widely. Thus, it is necessary to develop a rapid method to identify the producing area and authenticity of Pu-erh tea. Traditionally, the origins of tea have been partially judged by experts based on taste and aroma, but this is often unreliable [7]. The chromatographic fingerprint technique, widely used in traditional Chinese medicines for quality control, has been attracting attention because it emphasizes the systematic characterization of the composition, identification and the evaluation of sample stability [8]. In recent years, chromatogram fingerprint technology has been introduced into tea quality control research. Metabolic fingerprinting, which is based on modern separation techniques, can fully indicate the tea components and their relative proportions, and thus, has been widely used to identify the geographical origin and authenticity of various teas [9, 10]. Several chromatographic methods, including headspace solid-phase micro extraction (HS-SPME) combined with gas chromatography-mass spectrometry (GC-MS), high performance liquid chromatography (HPLC) and ultra-performance liquid chromatography tandem mass-spectrometry (UPLC-MS), have been used to explore the fingerprints of various teas [11–13]. As far as we know, no study has reported the ability to discriminate between sun-dried Pu-erh green teas from different tea mountains using chemical fingerprint similarity calculation software combined with principal component analysis (PCA) and cluster analysis (CA).

In this work, the volatile components of Pu-erh green teas were extracted and identified by HS-SPME combined with GC-MS. The chemical fingerprint similarity, combined with PCA and CA, were developed to evaluate the differences between Pu-erh green teas from two tea mountains (Jingmai and Wuliang). The objective of the current work was to develop an effective tool to identify the origins and authenticity of different Pu-erh green teas.

## Results and discussions

### Similarity analysis

The similarity analysis of volatile fingerprints was performed using computer-aided similarity evaluation software. Sixty-eight chromatographic peaks were recognized in the sun-dried Pu-erh green teas and 63 peaks were identified using NIST 08.L MS library and retention indices (RIs). Of these, 45 peaks were common to all of the Pu-erh green teas. The common peaks were confirmed by relative standard deviation (RSD %) values having relative retention time values of less than 1 %.

### Comparing the fingerprints of Pu-erh green teas from Jingmai and Wuliang mountains

**Fig. 1:**
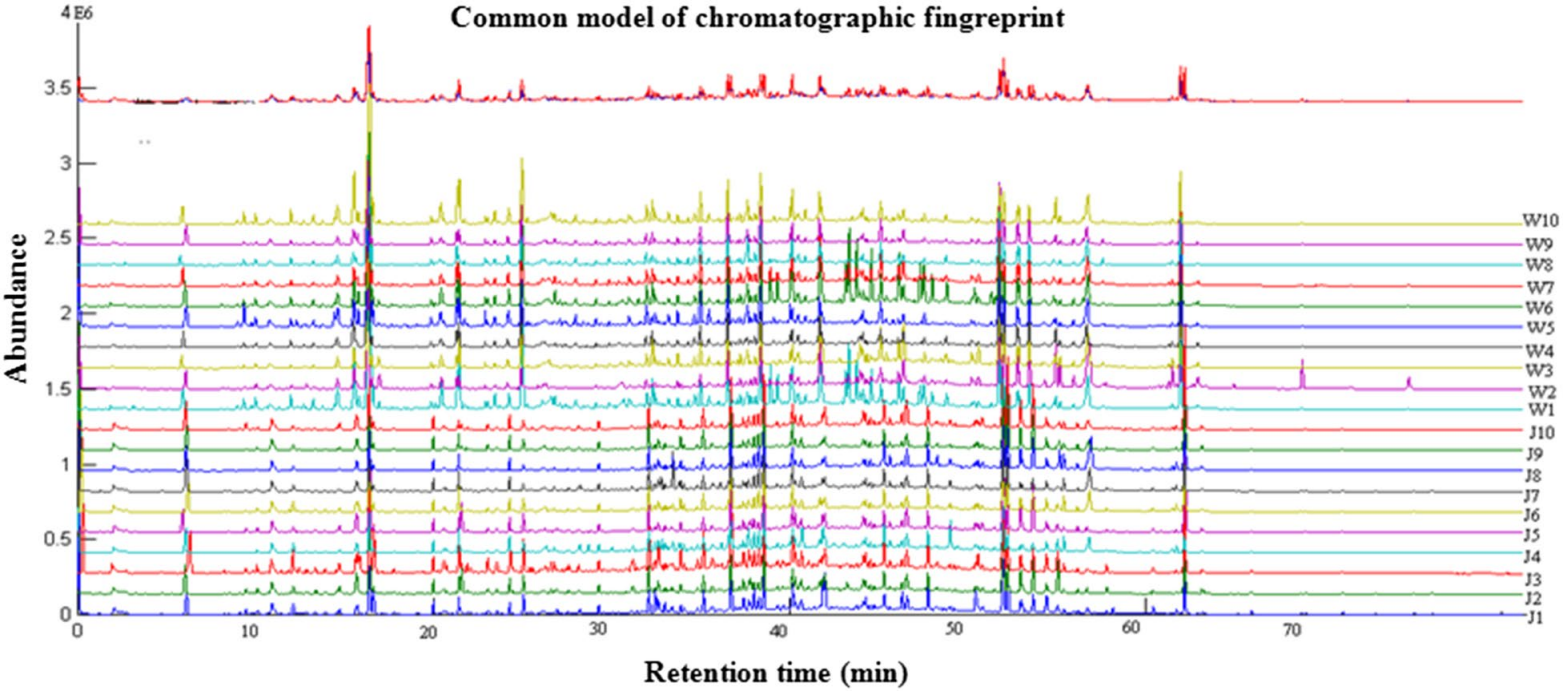
The overlapping GC-MS fingerprint plots of 20 Pu-erh green tea samples

The overlaid GC-MS chromatograms of 20 Pu-erh green teas from Jingmai and Wuliang mountain are shown in Fig. 1. All of the peaks were integrated using a computer-aided similarity evaluation system. As shown in Fig. 1, the volatile fingerprint information for these teas was abundant. A common reference chromatogram is usually regarded as the standardized characteristic fingerprint of the samples to be analyzed. Our reference chromatogram was automatically generated from the 20 overlaid GC-MS chromatograms by the computer-aided similarity evaluation system. The similarity values of all 20 inputted total ion chromatograms (TICs), relative to the reference chromatogram, can be calculated by correlation and congruence coefficients with median or averaged data [14, 15]. When the coefficient values are close to one, the two chromatographic fingerprints will show a high similarity. The similarities of all 20 Pu-erh green teas were calculated based their common model and the results are shown in Table 1.

**Table 1:** Evaluation of the similarity of 20 Pu-erh green tea samples using their common model of chromatographic fingerprints

Sample no.	Correlation coefficient		Congruence coefficient (R)	
	Median	Average	Median	Average
J1	0.6347	0.6741	0.7254	0.7525
J2	0.7110	0.7465	0.7743	0.8009
J3	0.5918	0.6542	0.6947	0.7382
J4	0.7544	0.7076	0.8278	0.7912
J5	0.6534	0.7082	0.7149	0.7580
J6	0.6028	0.6284	0.6920	0.7096
J7	0.5844	0.6141	0.6893	0.7083
J8	0.5931	0.6554	0.6880	0.7329
I9	0.5614	0.6502	0.6453	0.7133
J10	0.6515	0.6715	0.7293	0.7433
W1	0.6770	0.7901	0.7344	0.8230
W2	0.7012	0.7299	0.7553	0.7782
W3	0.8195	0.8075	0.8596	0.8510
W4	0.7935	0.7840	0.8237	0.8185
W5	0.7292	0.8262	0.7703	0.8480
W6	0.7086	0.8081	0.7580	0.8366
W7	0.7788	0.7947	0.8291	0.8411
W8	0.7489	0.7336	0.7973	0.7863
W9	0.6789	0.6942	0.7399	0.7518
W10	0.6877	0.7878	0.7385	0.8180

As seen in Fig. 1 and Table 1, the chromatograms of the 20 Pu-erh green tea samples were similar, and the similarities among them were relatively high. Thus, these data provided little information on the differences among the 20 teas from the two tea mountains. However, the high degree of similarity between them implies that these samples have similar compositions and aroma contents. Using the data presented in Table 1 and Fig. 1, the teas from the two tea mountains cannot be distinguished from one another.

### Construction of the typical GC-MS fingerprint of Jingmai Pu-erh green tea

The overlaid GC-MS chromatograms of the 10 Pu-erh green teas from the Jingmai mountain are shown in Additional file 1: Figure S1 (see supporting information). Forty-eight common peaks were found in these 10 Jingmai Pu-erh green teas, and the similarity analysis result based on their common model is shown in Table 2. The similarity value for the 10 Jingmai Pu-erh green teas was more than 0.7500, indicating a high degree of similarity in fingerprint characteristics among samples from the same tea mountain. However, when the chromatograms of the 10 Wuliang Pu-erh green teas were analyzed with the similarity evaluation software using the common model of 10 Jingmai Pu-erh green teas, their similarity value was significantly less (Table 2), implying that there were differences between the Jingmai and Wuliang mountain teas.

**Table 2:** Evaluation of the similarity of 20 Pu-erh green tea samples using Jingmai Pu-erh green tea common model of chromatographic fingerprints

Sample no.	Correlation coefficient		Congruence coefficient (R)	
	Median	Average	Median	Average
J1	0.7959	0.8705	0.8391	0.8985
J2	0.9059	0.9067	0.9237	0.9241
J3	0.7500	0.8086	0.8030	0.8504
J4	0.8181	0.8263	0.8606	0.8694
J5	0.8853	0.8708	0.9009	0.8878
J6	0.7766	0.7968	0.8187	0.8354
J7	0.7547	0.8009	0.7821	0.8435
J8	0.8290	0.8853	0.8617	0.9066
I9	0.8158	0.8710	0.8428	0.8869
J10	0.9575	0.9207	0.9649	0.9349
W1	0.2594	0.3101	0.4008	0.4469
W2	0.2853	0.3898	0.4139	0.5036
W3	0.2314	0.3259	0.3785	0.4598
W4	0.3382	0.3564	0.4799	0.4998
W5	0.2766	0.3615	0.3907	0.4654
W6	0.2620	0.2875	0.3913	0.4169
W7	0.2403	0.2565	0.3835	0.4020
W8	0.2314	0.2528	0.3681	0.3906
W9	0.2146	0.2619	0.3366	0.3804
W10	0.2570	0.2743	0.3902	0.4092

### Construction of the typical GC-MS fingerprint of Wuliang Pu-erh green tea

Using the same method, the typical GC-MS fingerprint of the 10 Wuliang Pu-erh green teas was constructed, and the overlaid GC-MS chromatograms are shown in Additional file 2: Figure S2 (see supporting information). Fifty-four common peaks were found in the 10 Wuliang Pu-erh green teas. The similarities among the 10 Wuliang Pu-erh green teas were calculated based on their common model as shown in Table 3. We also analyzed the chromatograms of the 10 Jingmai Pu-erh green teas with the similarity evaluation software using the common model of the 10 Wuliang Pu-erh green teas. The results (Table 3) showed that samples from same mountain were highly similar, their similarity value all more than 0.7523, indicating a stable aroma characteristic for samples from the same mountain. Interestingly, an obvious decrease in the similarities among the 10 Jingmai Pu-erh green tea samples was observed when using the common model of the 10 Wuliang Pu-erh green teas. This indicated that there were some differences between Pu-erh green teas from the Wuliang and Jingmai mountains. Thus, 20 Pu-erh green tea samples from the two tea mountains can be distinguished by their volatile fingerprints using the fingerprint similarity method.

**Table 3:** Evaluation of the similarity of 20 Pu-erh green tea samples using Wuliang Pu-erh green tea common model of chromatographic fingerprints

Sample no.	Correlation coefficient		Congruence coefficient (R)	
	Median	Average	Median	Average
W1	0.7944	0.8597	0.7527	0.8814
W2	0.8395	0.8341	0.8651	0.8610
W3	0.9633	0.9137	0.9698	0.9305
W4	0.9663	0.9093	0.9708	0.9228
W5	0.7876	0.8912	0.8189	0.9058
W6	0.7590	0.8977	0.7966	0.9128
W7	0.9624	0.9274	0.9691	0.9415
W8	0.9323	0.8751	0.9439	0.8970
W9	0.8512	0.8326	0.8753	0.8602
W10	0.7523	0.8681	0.7731	0.8873
J1	0.2594	0.3101	0.4008	0.4469
J2	0.2853	0.3898	0.4139	0.5036
J3	0.2314	0.3259	0.3785	0.4598
J4	0.3382	0.3564	0.4799	0.4998
J5	0.2766	0.3615	0.3907	0.4654
J6	0.2620	0.2875	0.3913	0.4169
J7	0.2403	0.2565	0.3835	0.4020
J8	0.2314	0.2528	0.3681	0.3906
J9	0.2146	0.2619	0.3366	0.3804
J10	0.2570	0.2743	0.3902	0.4092

### Methodology validation

The accuracy of the GC-MS analysis was evaluated by the continuous extraction and injection of the J1 sample five times under the same GC-MS conditions. The results indicated that the RSD values of the relative retention times of common peaks were less than 0.31 % and the RSD values of relative peak areas of common peaks were less than 2.01 %, thereby, validating the accuracy of the GC-MS analysis. The stability was determined at 0, 1, 2, 4, 8, 12, and 24 h by extracting J1 sample under the same GC-MS conditions. The results indicated that the RSD values of the relative retention times and relative peak areas of volatiles were less than 0.42 and 3.96 %, respectively, suggesting that the reasonable time length for sample analysis is within 24 h.

### Peak alignment and recognition by principal component analysis

**Fig. 2:**
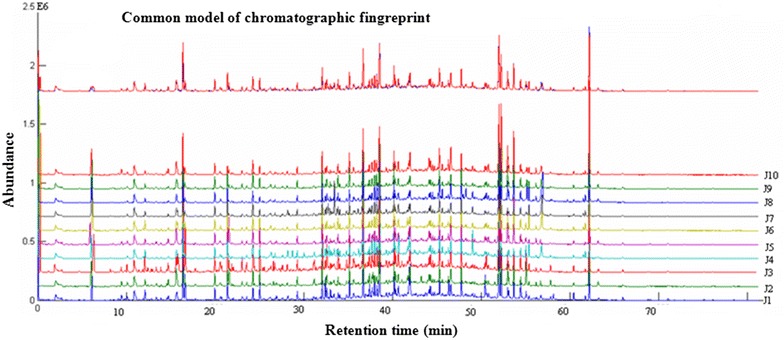
The results of the PCA-based chromatographic fingerprints of Jingmai and Wuliang Pu-erh green teas

A chromatographic fingerprint may contain a huge number of peaks, requiring chemometric methods to extract the desired information, such as common peaks, similarities among chromatograms, and projection and clustering plots of samples [16]. The computer-aided similarity evaluation software can perform whole spectrum recognition based on integrated fingerprint information and can directly determine relationships between samples in the two-dimensional projection cluster plot of the PCA. Prior to the runtime analysis of the PCA, the chromatographic fingerprints were preprocessed using wavelet smoothness. The PCA result based on all 20 TICs using fingerprint similarity evaluation software is shown in Fig. 2. The fingerprints were divided into two groups, and the group containing fingerprints 1–10 represented the Jingmai Pu-erh green teas and the other group, consisting of fingerprints 11–20, represented the Wuliang Pu-erh green teas. These results indicated that tea samples from different tea mountains have differences in volatile accumulation.

### Analysis of volatile compounds in Jingmai and Wuliang Pu-erh green teas

The volatiles of 20 Pu-erh green tea samples from the Wuliang and Jingmai mountain were isolated and identified using HS-SPME/GC-MS analysis. Table 4 lists the 63 identified volatile components. All 20 tea samples had very similar aroma compositions, mainly including alcohols, hydrocarbons (alkane, olefin, and aromatic hydrocarbon), and ketones. There were significant differences (*p* < 0.05) in the alcohol contents between the Wuliang (50.77 %) and Jingmai (29.81 %) Pu-erh green teas. The hydrocarbons contents showed little difference (*p* > 0.05), while the ketones were significantly different between the Wuliang and Jingmai Pu-erh green teas (*p* < 0.05).

**Table 4:** Volatile compounds and their relative contents of Jingmai and Wuliang Pu-erh green tea

No	RI^a^	Compound^b^	Relative percentage content^c^	
			Jingmai mountain (n = 10)	Wuliang mountain (n = 10)
1	957	Benzaldehyde	0.11 ± 0.10a	0.10 ± 0.07a
2	979	1-Octen-3-ol	0.44 ± 0.35a	0.65 ± 0.44a
3	985	6-Methyl-5-heptene-2-one	0.08 ± 0.12a	0.14 ± 0.09a
4	989	2-Pentyl-furan	0.52 ± 0.23a	0.63 ± 0.10a
5	997	α-Phellandrene	0.12 ± 0.12a	0.06 ± 0.08a
6	1011	α-Terpinene	0.12 ± 0.09a	0.06 ± 0.06a
7	1026	D-Limonene	1.12 ± 0.47a	0.64 ± 0.22b
8	1034	Benzyl alcohol	0.16 ± 0.11a	0.21 ± 0.13a
9	1037	(E)-3,7-Dimethyl-1,3,6-octatriene	0.11 ± 0.10a	0.20 ± 0.05b
10	1042	Phenyl acetaldehyde	0.11 ± 0.07a	0.21 ± 0.18b
11	1048	Ocimene	0.32 ± 0.09a	0.42 ± 0.13a
12	1056	γ-Terpinene	0.27 ± 0.11a	0.21 ± 0.06a
13	1068	(E)-2-Octen-1-ol	0.23 ± 0.23a	0.38 ± 0.32a
14	1072	Linalool oxide I	0.75 ± 0.25a	1.75 ± 0.29b
15	1087	Linalool oxide II	2.24 ± 0.80a	3.21 ± 0.96b
16	1098	Linalool	7.86 ± 2.44a	20.56 ± 3.47a
17	1100	Hotrienol	1.50 ± 1.30a	1.32 ± 0.48a
18	1110	Phenylethyl alcohol	0.07 ± 0.10a	0.65 ± 0.83b
19	1175	Linalool oxide IV	0.57 ± 0.23a	1.62 ± 0.32b
20	1178	Naphthalene	0.23 ± 0.14a	0.23 ± 0.17a
21	1188	α-Terpineol	2.43 ± 0.84a	2.16 ± 0.53a
22	1190	Methyl salicylate	0.99 ± 1.19a	2.91 ± 0.76a
23	1196	Safranal	0.29 ± 0.07a	0.35 ± 0.10a
24	1218	β-Cyclocitral	0.59 ± 0.20a	0.66 ± 0.24a
25	1228	Nerol	0.46 ± 0.15a	0.77 ± 0.17b
26	1256	Geraniol	1.58 ± 0.59a	5.41 ± 1.65b
27	1289	Indole	0.13 ± 0.22a	0.53 ± 0.34b
28	1300	Tridecane	0.17 ± 0.07a	0.18 ± 0.08a
29	1302	1-Methyl-naphthalene	0.40 ± 0.10a	0.18 ± 0.13b
30	1316	1,2,3-Trimethoxybenzene	0.56 ± 0.41a	0.44 ± 0.25a
31	1351	2,6-Dimethoxyphenol	0.15 ± 0.22a	0.32 ± 0.20a
32	1397	cis-Jasmone	0.86 ± 0.61a	1.30 ± 0.56a
33	1400	Tetradecane	1.48 ± 0.31a	0.64 ± 0.09b
34	1417	β-Caryophyllene	1.00 ± 1.57a	0.39 ± 0.22a
35	1428	α-Ionone	1.22 ± 0.29a	0.77 ± 0.18b
36	1455	Geranyl acetone	2.08 ± 0.42a	1.96 ± 0.46a
37	1487	β-Ionone	5.28 ± 1.45a	3.44 ± 0.64b
38	1500	Pentadecane	0.84 ± 0.30a	0.53 ± 0.13a
39	1506	Dibenzofuran	1.25 ± 0.77a	0.53 ± 0.09b
40	1508	α-Farnesene	0.30 ± 0.40a	1.19 ± 0.87b
41	1528	Dihydroactinidiolide	6.81 ± 1.53a	3.80 ± 0.55b
42	1554	Nerolidol	1.10 ± 0.25a	1.94 ± 0.69b
43	1572	Fluorene	1.63 ± 0.70a	0.88 ± 0.15a
44	1598	Cedrol	1.18 ± 0.65a	2.76 ± 0.98b
45	1600	Hexadecane	1.76 ± 0.62a	1.10 ± 0.29a
46	1653	α-Cadinol	1.06 ± 0.19a	0.83 ± 0.17b
47	1659	2,2′,5,5′-Tetramethyl-1,1′-biphenyl	0.70 ± 0.19a	0.32 ± 0.10a
48	1664	2-Methyl-hexadecane	0.50 ± 0.09a	0.11 ± 0.23a
49	1700	Heptadecane	0.97 ± 0.30a	0.82 ± 0.31a
50	1706	2,6,10,14-Tetramethyl pentadecane	2.35 ± 0.60a	1.45 ± 0.79a
51	1765	Anthracene	0.92 ± 1.17a	0.50 ± 0.18a
52	1800	Octadecane	0.69 ± 0.50a	0.30 ± 0.11a
53	1809	2,6,10,14-Tetramethyl hexadecane	0.89 ± 0.33a	0.64 ± 0.42a
54	1828	Isopropyl myristate	0.08 ± 0.12a	0.11 ± 0.12a
55	1840	Caffeine	9.10 ± 2.82a	5.07 ± 1.55a
56	1846	Phytone	4.58 ± 2.08a	2.31 ± 0.83a
57	1918	Farnesyl acetone	1.21 ± .32a	0.26 ± 0.24b
58	1927	Hexadecanoic acid methyl ester	1.16 ± 0.69a	0.51 ± 0.34b
59	1949	Isophytol	0.47 ± 0.19a	0.35 ± 0.11a
60	1975	Hexadecanoic acid	1.83 ± 1.79a	3.75 ± 1.47a
61	2093	Methyl linoleate	0.26 ± 0.23a	0.19 ± 0.09a
62	2099	Methyl linolenate	0.57 ± 0.34a	0.46 ± 0.28a
63	2122	Phytol	7.71 ± 4.14a	6.20 ± 1.93a
Alcohols			29.81a	50.77b
Hydrocarbons			16.89a	11.05a
Ketones			15.31a	10.18b
Esters			3.06a	4.18a
Aldehydes			1.10a	1.32a
Nitrogen compounds			9.23a	5.60a
Lactones			6.81a	3.80b
Others			4.31a	5.67a
Identified			86.52	92.57

^a^ *RI* retention indices as determined on HP-5MS column using the homologous series of n-alkanes (C_8_–C_40_)

^b^Compounds are listed in order of retention time

^c^The content of volatile compounds were represented as mean value ± standard deviation (mean ± SD), same letter in the same row indicates no significant differences (p < 0.05)

The identified volatiles in the Jingmai Pu-erh green teas mainly included caffeine, linalool, phytol, dihydroactinidiolide, β-ionone, phytone, α-terpineol, and 2,6,10,14-tetramethyl pentadecane, while the identified volatiles in the Wuliang Pu-erh green teas mainly included linalool, phytol, geraniol, caffeine, hexadecanoic acid, β-ionone, linalool oxide II, methyl salicylate, cedrol, and phytone. Because these two teas were produced using the same processing technology, it is not surprising that they had similar aroma compositions and contents. However, as seen in Table 4, some aroma components were significantly different between Jingmai and Wuliang Pu-erh green teas, especially some terpene alcohols and ketones, such as linalool and its oxides, hotrienol, nerol, geraniol, nerolidol, α-ionone, β-ionone, phytone, and farnesyl acetone. Volatile terpenoids, which generally have a green, fresh, or citrus-flavored fruit-juice, and a sweet and flowery aroma, are important for the tea’s quality [17, 18]. The sweet and floral aroma of linalool oxides are not from the oxidization of linalool, instead they come from the glycoside forms of linalool oxides in fresh tea leaves [19]. Ketone compounds were mainly biosynthesized by carotenoid. Carotenoid-derived volatile compounds can contribute to the woody fragrance of tea [20]. For example, β-ionone, which has a low odor threshold of 0.007 ppb, is a significant contributor to the flavor of green and black teas, and β-ionone can be produced either by enzymatic reactions during fermentation or by thermal degradation during the green tea manufacturing process [21, 22].

The characteristic volatile components of Pu-erh green teas may result from the different tea mountains and tea genotypes. Jingmai and Wuliang mountains have different environmental and geographical conditions, such as sunlight, rainfall, and soil type. Additionally, their tea trees may be different strains, which could result in different contents of aroma precursors during biosynthesis. Even if Pu-erh green teas have been treated by the same processing technology, the above mentioned factors may still result in differences in their aroma component contents. The biosynthetic pathways of different volatile components and their impact factors will be studied in the future.

### Principal component analysis of Jingmai and Wuliang Pu-erh green teas based on volatile components

**Fig. 3:**
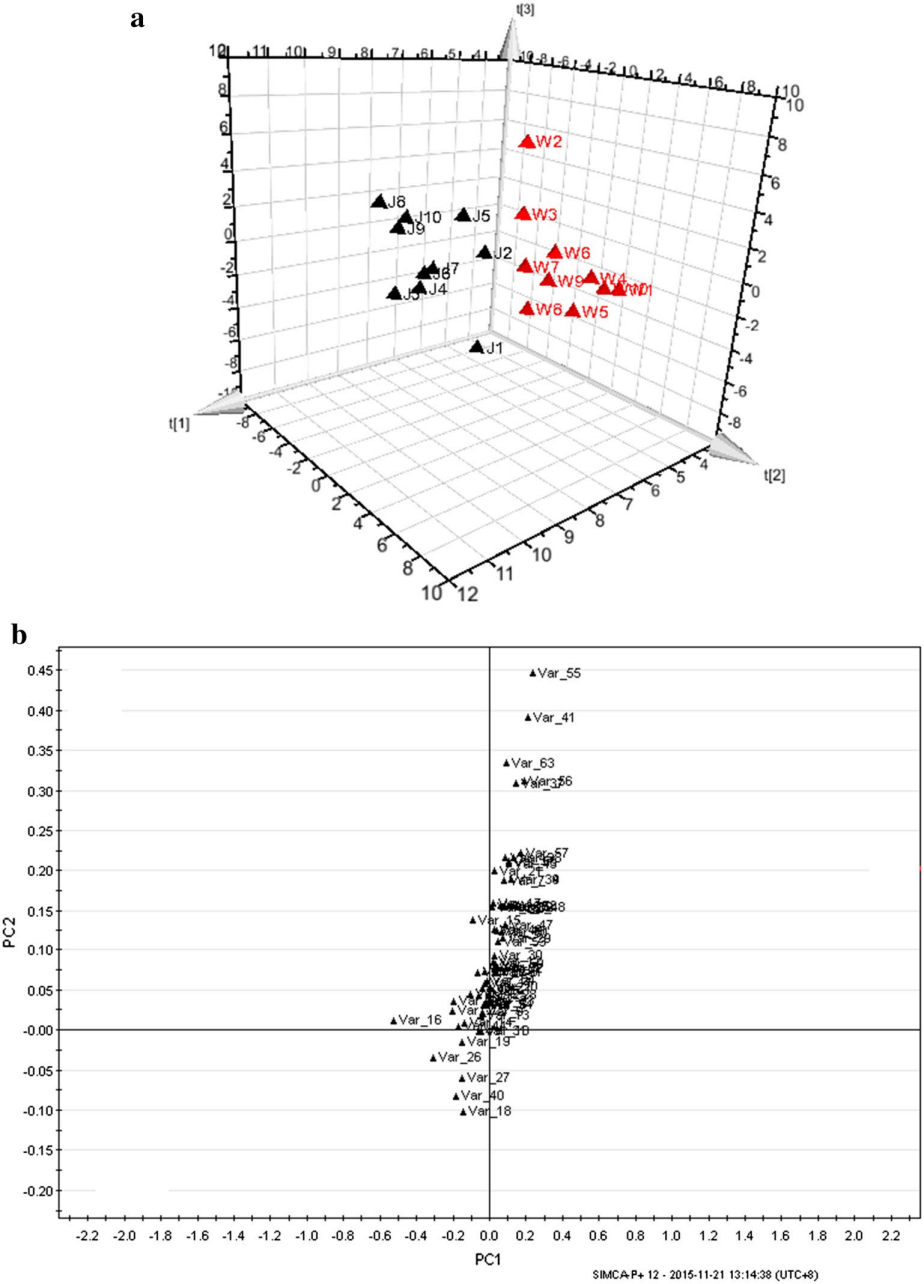
3D PCA score* plots* (**a**) and loading (**b**)* plots* derived from 63 volatile compounds in the 20 Pu-erh green tea samples

PCA is an unsupervised statistical method used to investigate the differences between sample groups by the reduction in the dimensions of the principal components [23–25]. PCA is frequently applied as a tool to exploratory data analysis [26]. In this work, PCA was applied to determine whether there were differences in the volatile patterns between Jingmai and Wuliang Pu-erh green teas. The identified volatiles (Table 4) in the 20 Pu-erh green tea samples were manually integrated and their relative percentages were analyzed using PCA. Prior to the runtime analysis of the PCA, the data were preprocessed by par scaling (the variables are centered and divided by the square root of their standard deviation). Figure 3a shows the score plot of the third principal components, PC_1_, PC_2_, and PC_3_, which represent 92.06 % of the total variability. Figure 3b shows the loading scatter plot, which establishes the relative importance of each variable, and it was used to study the relationships among variables and tea samples. As can be seen from Fig. 3a, the 20 Pu-erh tea green tea samples were successfully divided into two groups based on the relationships between the mountains (scores) and their volatile compound contents (loadings). Additionally, samples J2 and J5, and samples W2 and W3, were found in close proximity to each other. Figure 3b indicated that loadings V55 (caffeine), V41 (dihydroactinidiolide), V63 (phytol), V56 (phytone), V37 (β-ionone), V18 (phenylethyl alcohol), V40 (α-farnesene), V27 (indole), and V26 (geraniol) may have more influence on the ability to discriminate between samples from the two tea mountains than other volatile components. Overall, originating from different mountains results in the teas having some differences, while the same processing technology results in similarities among the volatile components.

### Cluster analysis

**Fig. 4:**
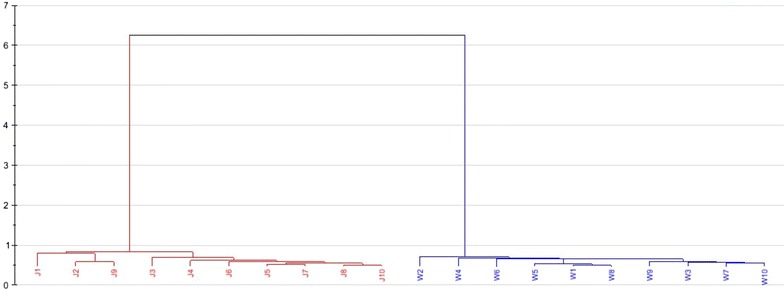
CA analysis based on the 63 volatile compounds of the 20 Pu-erh green tea samples

Like PCA, CA is also an unsupervised data analysis method that requires no prior knowledge of the test sample. CA is another method that was applied to extract information on the differences among different Pu-erh teas. It finds the dissimilarities among objects in a multidimensional space and divides all of the samples into groups (clusters) based on these dissimilarities [27, 28]. In the present study, all of the percentage quantitative data of the 63 volatile compounds were used in the CA model. The sample similarities were calculated based on the Euclidean distance [29], and Ward’s method was used as the amalgamation rule. The dendrogram result of the CA, indicating that these Pu-erh green tea samples were clustered into two groups, similar to the PCA results, are shown in Fig. 4.

An attempt has been made to recognize and differentiate Pu-erh green tea samples having different mountains of origin using HS-SPME extraction technique combined with GC-MS and multivariate statistical analyses. Based on the fingerprint similarity and PCA results, Pu-erh green tea samples from Jingmai and Wuliang mountain can be distinguished. This is likely related to different altitudes, climate conditions, geographical environments, ecology factors, tea varieties, cultivation conditions, and exogenous induction factors between these two tea mountains. However, the number of samples analyzed was relatively small, and the data analysis model was limited. To minimize the possibility of false results, future studies should include larger numbers of tea samples and employ more data processing methods. Nevertheless, the developed method can serve as a tool to distinguish between Pu-erh green teas of different origins, and the method can also be applied to other types of tea.

## Experimental

### Tea samples

Twenty sun-dried Pu-erh green teas, half from Jingmai mountain and half from Wuliang mountain were used in this work. All of these samples were produced in April 2014, and processed using the same technology.

### HS-SPME method

The solid-phase extraction coating (65 µm polydimethylsiloxane/divinylbenzene) was provided by Supelco (Bellefonte, PA, USA). The HS-SPME method was verified in our previous study [30]. The ground tea samples (2.0 g) were weighed and placed in 20 mL sealed headspace vials. After adding 4 g potassium chloride and 20 mL water, the vials were tightly sealed with a PTFA septum. The sample vials were equilibrated for 15 min in an 80 °C water bath shaking at 400 rpm, and then a fiber was exposed to the headspace over the sample for 60 min. Finally, the fiber was exposed in the GC injector for 3.5 min.

### GC-MS analysis

A 7890A GC-5975C MS system (Agilent Technologies, USA) was used for the separation and identification of tea volatile components. An HP-5MS capillary column (30 m × 0.25 mm × 0.25 µm film thicknesses) was used for the GC separation. Helium was used as the carrier gas at a flow rate of 1 mL min^−1^. The injector temperature was 250 °C and equipped with a split less injector. The employed temperature program had the following settings: an initial temperature of 50 °C (held for 1 min), increased to 210 °C at 3 °C min^−1^ (held for 3 min); and then increased to 230 °C at 15 °C min^−1^. The mass spectrometer was operated in an electron-impact mode of 70 eV. The mass scan range was 35–500 amu. The temperatures of the interface, ion source and quadrupole were 280, 230 and 150 °C, respectively. The solvent delay time was 2.8 min.

### Data processing

The volatile compounds were identified by comparing their mass spectra with those of the mass spectra libraries NIST 08 and Wiley 7. Additionally, a series of n-alkanes (C_8_–C_40_) were run under the same chromatographic conditions to calculate the RIs of detected compounds by comparing them with the RIs provided by the NIST Chemistry Web Book (http://webbook.nist.gov/chemistry/) and in previous literature [31–36].

The PCA and CA were performed using the SIMCA-P12 package (Umetrics, Umea, Sweden). The algorithms for the PCA and CA were from the SIMCA toolbox and were operated in SIMCA-P12. In addition, the fingerprint similarity analysis was performed using the traditional Chinese medicine fingerprint similarity evaluation system (Version 1.0 for Windows, Central South University, China), which was provided by the Research Center for Modernization of Chinese Medicines of the Central South University. The correlation (R_1_) and congruence (R_2_) coefficients between two fingerprints were calculated with median or average data and were then expressed as follows:

1 $$R_{1} = \frac{{\sum\nolimits_{i = 1}^{n} {(x_{i} - \bar{x})(y_{i} - \bar{y})} }}{{\sqrt {\sum\nolimits_{i = 1}^{n} {(x_{i} - \bar{x})^{2} \sum\nolimits_{i = 1}^{n} {(y_{i} - \bar{y})} } } }}(i = 1,2,3, \ldots ,n),$$

2 $$R_{2} = \frac{{\sum\nolimits_{i = 1}^{n} {x_{i} y_{i} } }}{{\sqrt {\sum\nolimits_{i = 1}^{n} {(x_{i} )^{2} \sum\nolimits_{i = 1}^{n} {(y_{i} )^{2} } } } }}(i = 1,2,3, \ldots ,n),$$

″x and y were the median values, the Eq. (2) is the famous cosine measure; it produces exactly the same values as Eq. (1) if the data are centered. Significant differences were determined by *t* test analyses of independent samples using the SPSS statistical package (version 17.0 for Window, SPSS, Inc., Chicago, USA).

## Conclusions

The volatile profiles of Pu-erh green teas from different mountains of origin (Jingmai and Wuliang) were evaluated by combining HS-SPME/GC-MS with multivariate statistical methods. There were some differences between Jingmai and Wuliang Pu-erh green teas, and it was possible to divide these samples into two groups that corresponded to their mountain origin using GC-MS information. Sixty-three volatiles were identified, with alcohols and hydrocarbons being the major aroma components in all 20 tea samples. Some aroma components exhibited significant differences between depending on the tea mountain origin, especially some terpene alcohol compounds and ketones. In conclusion, the combination of the GC-MS analysis with PCA and CA can successfully discriminate between mountain origins of Pu-erh green teas and, therefore, can be further applied to identify and authenticate Pu-erh green teas.

## Additional files

10.1186/s13065-016-0159-y The overlapping plots of the GC-MS fingerprints for 10 Jingmai Pu-erh green tea samples.
10.1186/s13065-016-0159-y The overlapping plots of the GC-MS fingerprints in 10 Wuliang Pu-erh green tea samples.
