# Supplementary figures and images for: Comparative analysis of volatiles difference of Yunnan sun-dried Pu-erh green tea from different tea mountains: Jingmai and Wuliang mountain by chemical fingerprint similarity combined with principal component analysis and cluster analysis
Source: Chem Cent J. 2016 Mar 10;10:11. doi: 10.1186/s13065-016-0159-y (PMC4785618; doi:10.1186/s13065-016-0159-y)

Figure S1 The overlapping plots of the GC-MS fingerprints for 10 Jingmai Pu-erh green tea samples

**
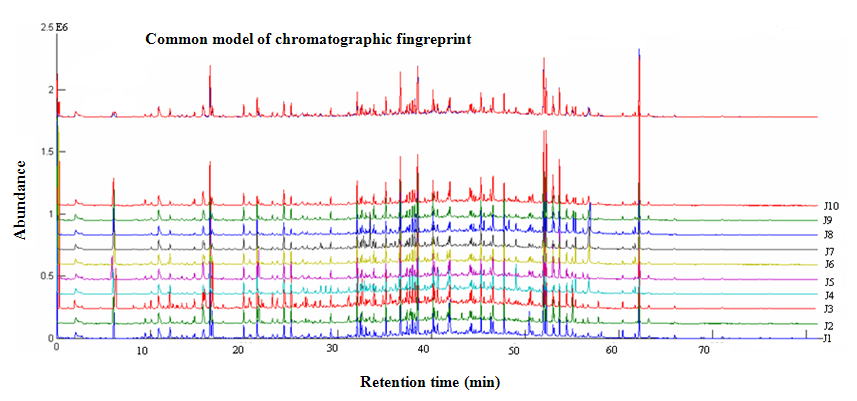
**

Supplement: Supplementary file 1 — 10.1186/s13065-016-0159-y The overlapping plots of the GC-MS fingerprints for 10 Jingmai Pu-erh green tea samples. [file 13065_2016_159_MOESM1_ESM.docx]

Figure S2 The overlapping plots of the GC-MS fingerprints in 10 Wuliang Pu-erh green tea samples


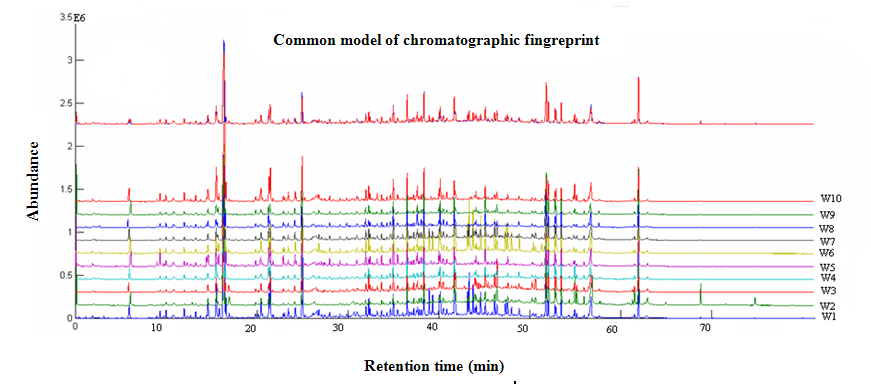

Supplement: Supplementary file 2 — 10.1186/s13065-016-0159-y The overlapping plots of the GC-MS fingerprints in 10 Wuliang Pu-erh green tea samples. [file 13065_2016_159_MOESM2_ESM.docx]
